# The impact of AIM2 inflammasome-induced pyroptosis on acute gouty arthritis and asymptomatic hyperuricemia patients

**DOI:** 10.3389/fimmu.2024.1386939

**Published:** 2024-07-19

**Authors:** Jiyan Chu, Jing Tian, Ping Li, Diyu Fu, Lin Guo, Rui Sun

**Affiliations:** ^1^ Department of Rheumatology, General Hospital of Northern Theater Command, Shenyang, Liaoning, China; ^2^ Graduate School, Dalian Medical University, Dalian, Liaoning, China; ^3^ Department of Orthopedics, General Hospital of Northern Theater Command, Shenyang, Liaoning, China

**Keywords:** absent in melanoma 2(AIM2), pyroptosis, caspase-1, gasdermin D (GSDMD), acute gouty arthritis, asymptomatic hyperuricemia

## Abstract

**Objective:**

This study aimed to evaluate the role of absent in melanoma 2 (AIM2) inflammasome-mediated pyroptosis in the pathogenesis of acute gouty arthritis (AGA) and asymptomatic hyperuricemia(AHU).

**Methods:**

A cohort of 30 AGA patients, 30 AHU individuals, and 30 healthy controls (HC) was assembled. Demographic and biochemical data, along with blood samples, were collected. Serum double-stranded DNA (dsDNA) levels were quantified using a fluorescent assay. Transcriptomic and proteomic analysis of AIM2, Caspase-1, GSDMD, IL-1β, and IL-18 in peripheral blood mononuclear cells was performed using qRT-PCR and Western blot. Enzyme-linked immunosorbent assay (ELISA) was employed to measure serum IL-1β and IL-18. Spearman correlation analysis was utilized to assess relationships between variables.

**Results:**

Both AGA and AHU groups demonstrated elevated metabolic indicators and serum levels of dsDNA, IL-1β, and IL-18 compared to the HC group. AGA patients exhibited higher inflammatory markers than the AHU group. In the AGA group, there was a significant increase in the mRNA and protein levels of AIM2, Caspase-1, GSDMD, IL-1β, and IL-18 (P<0.05 to P<0.001). The AHU group showed higher AIM2, Caspase-1, GSDMD, and IL-18 mRNA levels than the HC group (P<0.001 to P<0.01), with a non-significant increase in AIM2, GSDMD, and IL-1β proteins (P>0.05). In contrast, Caspase-1 and IL-18 proteins were significantly higher in the AHU group (P<0.05). Notable correlations were observed between AIM2 protein expression and levels of Caspase-1 and GSDMD in both AGA and AHU groups. In the AGA group, AIM2 protein correlated with IL-1β, but not in the AHU group. The AIM2 protein in the AHU group was positively associated with IL-18, with no such correlation in the AGA group.

**Conclusion:**

AIM2 inflammasome may play a role in the inflammatory processes of AGA and AHU and that its activation may be related to the pyroptosis pathway.

## Introduction

Gout and asymptomatic hyperuricemia (AHU) are impacting individuals worldwide, with a growing prevalence among younger people (1, 2). Acute gout arthritis (AGA) is an inflammatory disorder characterized by hyperuricemia and the deposition of monosodium urate (MSU) crystals in joints and tissues (3). Patients with AGA have episodes of severe inflammation, swelling, and pain. Without appropriate treatment, chronic gout can lead to cardiovascular disease and renal impairment (1, 3). In hyperuricemia, serum uric acid levels exceed the saturation threshold. The condition is known as asymptomatic hyperuricemia when it occurs in the absence of inflammation caused by monosodium urate crystals (4, 5). Asymptomatic hyperuricemia, traditionally considered a metabolic abnormality linked to gout and kidney stones, is now recognized as a significant risk factor for hypertension, adiposity, diabetes mellitus, hepatic steatosis, chronic nephropathy, and cardiometabolic diseases (5, 6). As safe and effective medications for AGA treatment are lacking, and the necessity of urate-lowering therapy for individuals with asymptomatic hyperuricemia is debatable, there is an urgent need to investigate the etiology of AGA and silent hyperuricemia.

Pyroptosis is a type of inflammatory programmed cell death, first proposed by Brennan and Cookson in 2001 (7). Unlike necrosis and apoptosis, it induces cellular membrane disruption and the secretion of pro-inflammatory cytokines, which amplify the inflammatory response (8). In individuals suffering from gout, the body activates innate immune cells by recognizing MSU through endogenous damage associated molecular patterns (DAMPs), which participate in pyroptosis (9). Pyroptosis is known to occur through three main pathways: the canonical pathway, the non-canonical pathway, and the Caspase-3 mediated pathway (10). The canonical pathway is mediated by inflammasomes and Caspase-1. Upon stimulation by PAMPs or DAMPs, the inflammasomes activate Caspase-1, promoting the maturation and release of pro-inflammatory cytokines like interleukin-1β (IL-1β) and IL-18, as well as the activation of gasdermin D (GSDMD). The activated effector molecule GSDMD forms non-selective pores on the cell membrane, leading to the loss of its integrity (11). Various inflammatory factors are released through these pores, while extracellular substances enter the cell, accelerating cell swelling and organelle damage, ultimately resulting in cell rupture.

Inflammasomes, composed of pattern recognition receptors (PRRs), apoptosis-associated speck-like protein containing a CARD (ASC), and inactive pro-caspase-1, are multiprotein complexes found in the cytoplasm (12). They can only function after they are activated. PRRs serve as the initial signal to induce transcription and synthesis of inactive pro-IL-1β and pro-IL-18. Inflammasomes then act as the second signal to convert pro-caspase-1 to active Caspase-1 (13). The PRRs of inflammasomes mainly include members of the nucleotide-binding oligomerization domain-like receptor (NLR) family and the absent in melanoma 2 (AIM2)-like receptor (ALR) family (14).

Absent in melanoma 2 (AIM2) constitutes a component of the AIM-like receptor (ALR) family, which contains an N-terminal pyrin domain (PYD) and a C-terminal domain that belongs to a group known as hematopoietic, interferon-inducible, nuclear proteins with a 200-amino-acid repeat (HIN-200). This receptor helps recognize cytosolic DNA via its HIN-200 region, which stimulates the adaptor protein ASC to facilitate the oligomerization and subsequent proteolytic maturation of pro-caspase-1 (15, 16). AIM2 is an intracellular DNA sensor that identifies genomic material originating from several sources, such as pathogenic bacteria, viral entities, genotoxic stress from ionizing radiation, and endogenously derived DNA disseminated via exosomal mechanisms (17–21). Researchers have found through experiments that the oligomeric assembly kinetics of the AIM2 inflammasome depends on the chain length of double-stranded DNA (dsDNA). The oligomerization is optimally induced by dsDNA that is 80–200 base pairs in length (22, 23). In the context of autoimmune diseases, such as systemic lupus erythematosus (SLE), rheumatoid arthritis (RA), and Sjögren’s syndrome (SS), dysregulated hyperactivation of the AIM2 inflammasome pathway may occur due to the continuous accumulation of self-derived DNA substrates (24–26). However, only a few studies have investigated the role of the AIM2 inflammasome in AGA. Therefore, in this study, we evaluated the expression of AIM2, Caspase 1, and GSDMD in PBMCs of patients diagnosed with AGA and AHU, estimated the levels of the inflammatory cytokines IL-1β and IL-18 in serum, hope to provide new insights into the involvement of the AIM2 mediated pyroptosis pathway in the development of AGA and strategies for treating gout.

## Materials and methods

### Participants and samples

Data were collected from patients attending outpatient clinics at the General Hospital of Northern Theater Command between December 2021 and August 2022. The study included 90 male participants aged between 18 and 70 years. Among them, 30 individuals were diagnosed with acute gouty arthritis based on the 2015 ACR/EULAR gout classification criteria and assigned to the AGA group (27). Another 30 individuals were diagnosed with asymptomatic hyperuricemia based on the hyperuricemia criteria (4) and assigned to the AHU group. Finally, 30 healthy participants who visited the medical examination center for a physical examination were assigned to the control group. Care was taken to match the age and gender distribution among the three groups.

The study excluded the following patients: (1) individuals with acute purulent arthritis, traumatic arthritis, and rheumatoid arthritis; (2) those with other crystal-related diseases like calcium pyrophosphate deposition disease; (3) patients with mental disorders, abnormal liver and kidney function, and cardiovascular and cerebrovascular diseases; (4) individuals with secondary gout; (5) pregnant women or those with malignancy.

All procedures used in the study were approved by the Medical Ethics Committee of the General Hospital of Northern Theater Command. Written informed consent was obtained from each participant. Total RNA and protein were extracted from blood samples stored at –80°C for subsequent analyses.

### Hematology analysis and determination of biochemical parameters

Peripheral venous blood (8 mL) was collected from the participants and processed by a hematology analyzer to quantify the white blood cell (WBC) count, hemoglobin (Hb) level, and platelet count (PLT). An automated biochemical analyzer was used to determine the levels of fasting blood glucose (FBG), uric acid (UA), triglycerides (TG), total cholesterol (TC), alanine aminotransferase (ALT), aspartate aminotransferase (AST), serum creatinine (Scr), blood urea nitrogen (BUN), erythrocyte sedimentation rate (ESR), and C-reactive protein (CRP). Creatinine clearance rate (Ccr) was calculated using the equation Ccr = (140-age) × body weight (kg)/72×Scr (mg/dL).

### Fluorescent quantification for determining serum dsDNA levels

The serum dsDNA was quantified using the Quant-iT PicoGreen dsDNA Kit (Invitrogen, USA), following the manufacturer’s instructions. Briefly, 90 µL of TE buffer was added to each well of a 96-well plate, followed by 10 µL of serum. Next, 100 µL of PicoGreen solution diluted 200 times with TE buffer was added to each well. The samples were incubated at room temperature for 10 min in the dark. A BioTek Synergy2 multifunctional microplate reader was used to detect and calculate the concentration of dsDNA under the excitation light at 480 nm and emission light at 520 nm.

### Total RNA isolation and real-time PCR analysis

Blood samples were processed using the Whole Blood Total RNA Kit (IVDSHOW, China) to extract total RNA according to the manufacturer’s instructions. The RNA was then used to synthesize cDNA using a reverse transcription kit (TaKaRa, Japan). Next, 1uL of the synthesized cDNA was used to measure the mRNA expressions of *AIM2*, *Caspase-1*, and *GSDMD* with specific primers as templates. The primers used in the amplification of the target mRNAs are shown below: *AIM2*: forward: 5′-AGCAAGATATTATCGGCACAGTG-3′, reverse: 5′-GTTCAGCGGGACATTAACCTT-3′; *Capase-1*: forward: 5′-TTTCCGCAAGGTTCGATTTTCA-3′, reverse: 5′-GGCATCTGCGCTCTACCATC-3′.

TACCATC-3′; *GSDMD*: forward: 5′-GTGTGTCAACCTGTCTATCAAGG-3′, reverse: 5′-CATGGCATCGTAGAAGTGGAAG-3′.

CATCGTAGAAGTGGAAG-3′; *IL-18*: forward: 5′-TTCAAGACCAGCCTGACCAAC-3′, reverse: 5′-GCTCACCACAACCTCTACCTCC-3′; *IL-1β*: forward: 5′-TGAGCTCGCCAGTGAAATGAT-3′, reverse: 5′-TGCTGTAGTGGTGGTCGGAG-3′; *GAPDH*: forward: 5′-ACAACTTTGGTATCGTGGAAGG-3′.

AAGG-3′, reverse: 5′-GCCATCACGCCACAGTTTC-3′. The analysis was performed using the Applied Biosystems 7500 Real-Time PCR system (Applied Biosystems, USA). The conditions used for thermal cycling were heating at 95°C for 5 min, followed by 40 cycles of denaturation at 95°C for 15s, and annealing at 60°C for 34s. The expression levels of *AIM2*, *Caspase-1*, and *GSDMD* mRNA were analyzed using the 2–ΔΔCT method after normalized by the expression of glyceraldehyde-3-phosphate dehydrogenase (*GAPDH*).

### Western blotting

PBMCs from various groups were lysed using a specific lysis buffer for Western blotting and immunoprecipitation. Protein concentrations were measured by the BCA assay kit (abs9232, Absin, China) and normalized. Next, proteins were separated by SDS-PAGE on either 10% or 12% gels (PG112 and PG113, Epizyme Scientific, China) according to their molecular weight and transferred to a 0.45 µm PVDF membrane. The proteins on the membrane were blocked with 5% non-fat milk for two hours at room temperature, then rinsed three times with tris-buffer saline tween (TBST) for 5 min. After rinsing, the protein on the membrane was incubated with primary antibodies against AIM2 (abs125828, rabbit polyclonal, 1:500, Absin, China), Caspase-1 (#3866, rabbit monoclonal, 1:1000, cell signaling technology, Danvers, MA), GSDMD (P57764, rabbit polyclonal, 1:500, Bioss, China), IL-1β (#12703, rabbit monoclonal, 1:1000, cell signaling technology, Danvers, MA), IL-18 (#54943, rabbit monoclonal, 1:1000, cell signaling technology, Danvers, MA), or GAPDH (GB15004, rabbit monoclonal, 1:2000, Servicebio, China) at 4°C on a shaker overnight. After thoroughly washing with TBST, the protein on the membranes was incubated with HRP-conjugated goat anti-rabbit IgG antibodies (GB23303, 1:3,000, Servicebio, China) at room temperature for 2 hours. The protein bands were visualized using an ECL chemiluminescence kit (abs920, Absin, China) and imaged with a GE AI 680 ultra-sensitive multifunctional imaging system. Image J software was used to analyze band intensities and calculate relative protein expression levels.

### ELISA

The concentrations of IL-18 and IL-1β in the cell supernatants were quantified using a human enzyme-linked immunosorbent assay kit (No:0139H1, Meimian, China; No: 0181H1, Meimian, China), following the manufacturer’s instructions. Duplicate samples were analyzed. The optical density (OD) values of the participants’ peripheral blood serum at 450 nm were measured using a Rayto RT-6100 microplate reader. Standard curves were generated by plotting the absorbance values against the graded concentrations of the standards provided with the kits. Positive and negative controls were simultaneously analyzed on the same plate.

### Statistical analysis

SPSS 23.0 was used for statistical analysis, and GraphPad Prism 10.1.2 was employed to generate graphs. Quantitative data following a normal distribution were presented as mean ± standard deviation ( $\bar{\text{x}}$ ± s), and intergroup differences were assessed using Student’s t-test. Quantitative data not conforming to a normal distribution were presented as Median (*P*25, *P*75), and intergroup differences were evaluated using the non-parametric Mann-Whitney U test. Intergroup differences were determined using one-way ANOVA followed by Tukey’s *post-hoc* test. Spearman correlation analyses were conducted to assess the relationship between variables. A statistically significant difference was defined as *P* < 0.05.

## Results

### Clinical characteristics and laboratory indices of the three groups

The clinical and laboratory characteristics of the three groups are summarized in **Table 1**. In contrast to the HC group, both the AGA and AHU groups exhibited significantly elevated levels of body mass index (BMI), uric acid (UA), total cholesterol (TC), triglyceride (TG), and platelet count(PLT). There were no statistically significant distinctions between the AGA and AHU groups. These findings suggest that gout/hyperuricemia is a contributory factor to metabolic syndrome, often accompanied by obesity, hyperlipidemia, and an inflammatory state. The AGA group exhibited significantly higher levels of white blood cells (WBC), erythrocyte sedimentation rate (ESR), and C-reactive protein (CRP) compared to the AHU group, aligning with the characteristic features of an acute gout attack. There were no significant differences in age, blood glucose (BG), alanine aminotransferase (ALT), aspartate aminotransferase (AST), and blood urea nitrogen (BUN) among the three groups. The level of creatinine clearance (Ccr) was higher in both the AGA and AHU groups than the healthy control group, indicating gout/hyperuricemia associated with increased renal blood flow, perfusion, and compensatory renal function.

**Table 1:** Clinical characteristics and laboratory indices of the three groups.

Items	AGA Group (n = 30)	AHU Group (n = 30)	HC Group (n = 30)
Age (year)	33.00 (26.00,39.00)	33.50 (30.50,37.25)	34.50 (28.75,46.50)
BMI (kg/m^2^)	28.18 ±2.93^##^	27.40 (25.63,28.40)^##^	23.89 ±2.27
WBC (×10^9^/L)	8.85 ±1.74^##^**	6.68 ±1.63	5.80 ±1.21
PLT (×10^9^/L)	276.03 ±50.67^##^	247.53 ±43.30^#^	226.17 ±46.58
ESR (mm/h)	17.70 ±10.94^##^**	3.00 (2.00,6.25)	2.00 (2.00,2.00)
CRP (mg/L)	14.35 (6.85,28.40)^##^**	2.90 (2.90,2.90)	2.90 (2.90,2.90)
UA (µmol/L)	535.37 ±97.02^##^	498.50 (466.00,549.25)^##^	355.10 ±38.35
FBG (mmol/L)	5.31 (5.00,5.66)	5.37 (5.16,5.53)	5.21 ±0.39
TC (mmol/L)	5.29 ±0.86^##^	4.90 (4.59,5.51)^##^	3.67 (3.36,4.48)
TG (mmol/L)	1.83 (1.41,2.76)^##^	2.01 (1.09,3.30)^##^	0.76 (0.65,1.41)
ALT (U/L)	28.50 (21.75,59.25)	26.50 (18.75,37.75)	25.50 (16.75,29.25)
AST (U/L)	22.00 (18.00,34.00)	20.50 (16.75,27.25)	20.50 (18.75,24.25)
BUN (mmol/L)	4.00 (4.00,5.00)	5.00 (4.38,6.00)	5.00 (4.83,6.00)
Scr (µmol/L)	92.53 ±17.00^##^*	84.10 ±9.42	82.83 ±9.19
Ccr (mL/min)	125.89 ±31.22^#^	127.81 (109.72,140.92)^#^	109.34 ±18.32

^#^P<0.05, ^##^P<0.01 vs. HC group; *P<0.05, **P<0.01 vs. AHU group.

### Serum dsDNA levels of the participants

**Figure 1:**
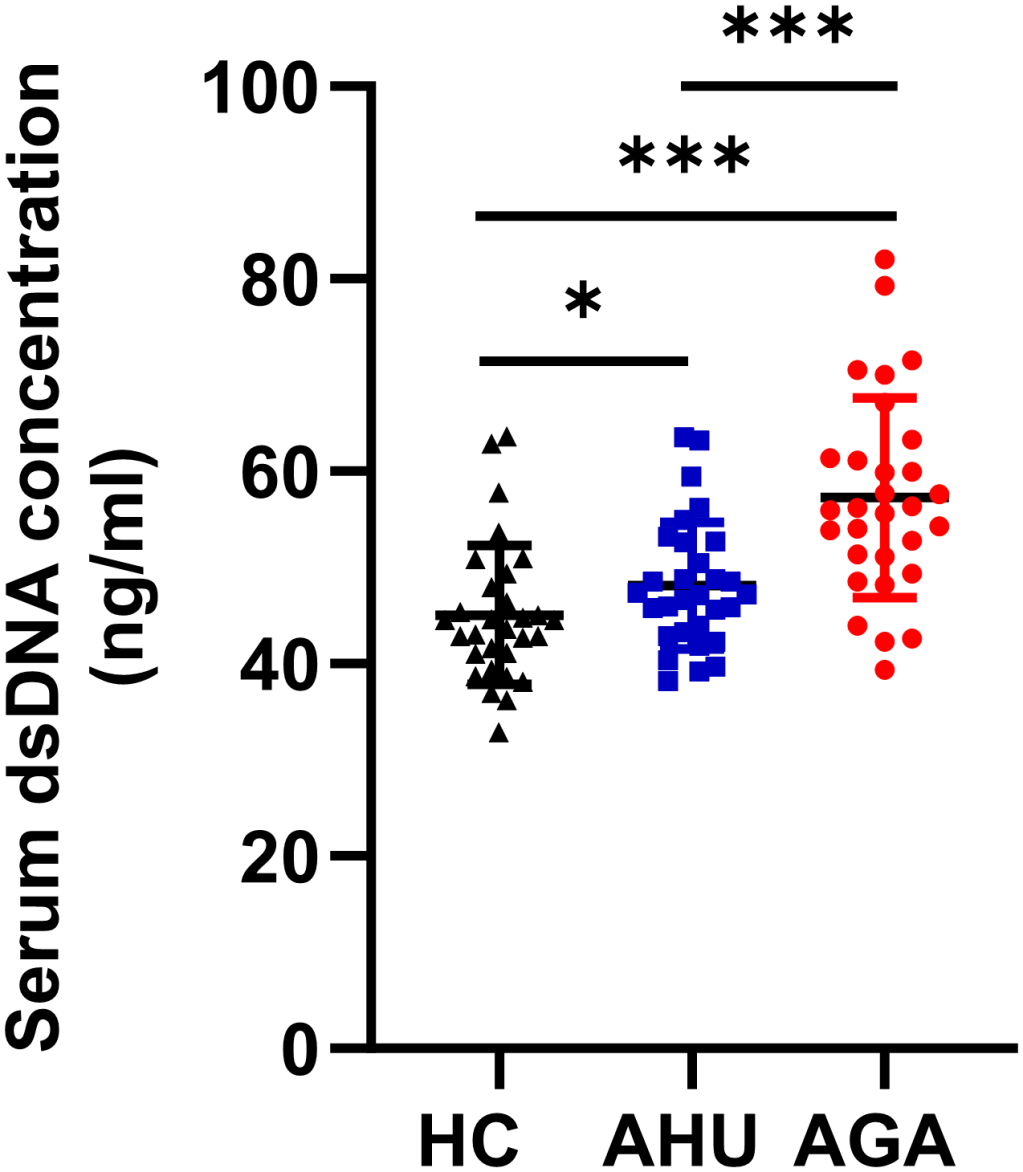
Serum dsDNA concentration of the three groups. Each cohort comprised 30 specimens. Serum levels of dsDNA detected by fluorescent quantification were most elevated in the AGA group, succeeded by the AHU group, with both groups exhibiting higher concentrations than the healthy control group. Comparative analyses between groups demonstrated statistically significant variances. *P < 0.05, ***P < 0.001.

The serum dsDNA level was significantly higher in the AGA group compared to both the AHU and HC groups, with a highly significant statistical difference (*P* < 0.001). In addition, the AHU group demonstrated a modest elevation in serum dsDNA concentration compared to the HC group, showing a statistically significant variance (*P* < 0.05), as illustrated in **Figure 1**.

### Relative level of expression of the mRNAs of *AIM2*, *Caspase 1*, *GSDMD*, *IL-1β*, and *IL-18* in PBMCs of the three groups

**Figure 2:**
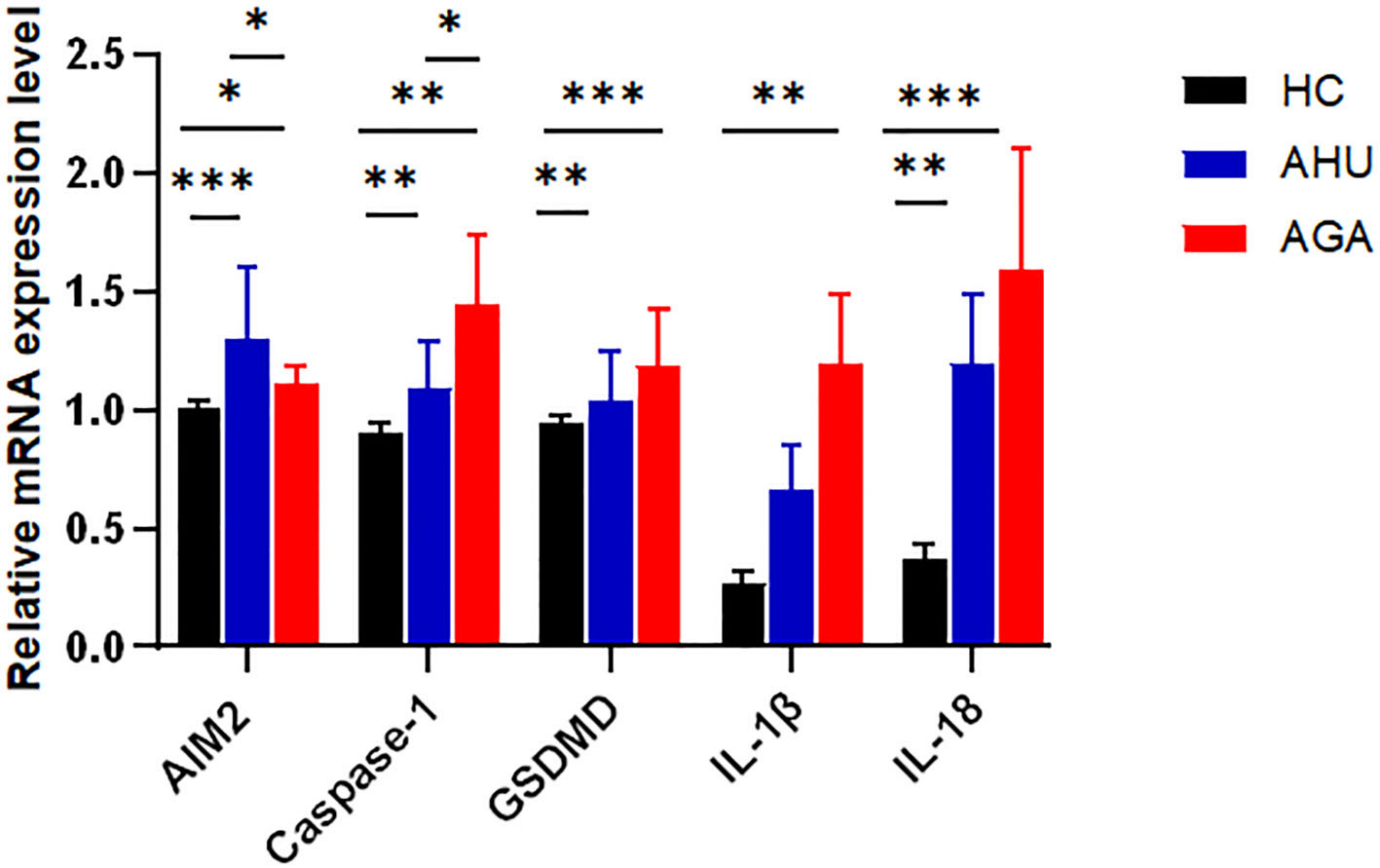
The mRNA expression levels of *AIM2, Caspase-1, GSDMD, IL-1β*, and *IL-18* relative to *GAPDH* in PBMCs were determined by real-time PCR in the three groups. Compared with the HC group(n=26), the AGA group (n=26) demonstrated elevated mRNA expression levels of *AIM2, Caspase-1, GSDMD, IL-1β*, and *IL-18*; similarly, the AHU group (n=26) exhibited increased expression levels of *AIM2, Caspase-1, GSDMD*, and *IL-18* mRNAs when compared to the HC group; the level of *AIM2* in the AHU group is higher than the AGA group, and all these differences were statistically significant. *P<0.05, **P < 0.01, ***P < 0.001.

As shown in **Figure 2**, compared with the HC group, the levels of *AIM2*, *Caspase-1*, *GSDMD*, *IL-1β*, and *IL-18* mRNAs expression were higher in the AGA group, and the difference was statistically significant (*P*<0.05, *P*<0.01, *P*<0.001, *P*<0.01, *P*<0.001). The levels of *AIM2*, *Caspase-1*, *GSDMD*, and *IL-18* mRNA expression were higher in the AHU group than in the HC group, with significant differences (*P*<0.001, *P*<0.01, *P*<0.01, *P*<0.01). The relative mRNA expression levels of *IL-1β* was also slightly higher in the AHU group than in the HC group, however, without statistically significant difference (*P*>0.05). The level of *Caspase-1* mRNA expression was higher in the AGA group than that in the AHU group, while *AIM2* mRNA expression in the AHU group higher than that in the AGA group, and the differences were all significant(*P*<0.05 for both).

### Relative level of proteins expression of AIM2, caspase 1, GSDMD, IL-1β, and IL-18 in the PBMCs of the three groups

**Figure 3:**
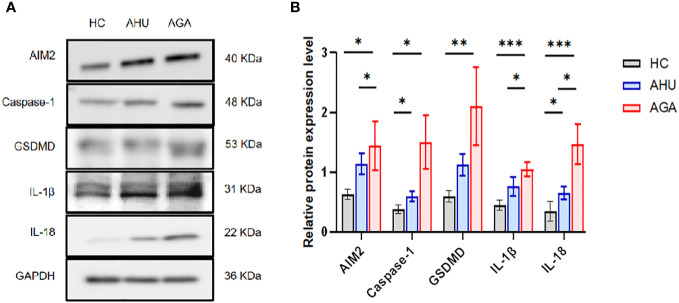
The relative expression levels of AIM2, Caspase-1, GSDMD, IL-1β, and IL-18 protein in the PBMCs of the three groups. **(A)** Western blot analysis was conducted to measure the protein expression of AIM2, Caspase-1, GSDMD, IL-1β, and IL-18 relative to GAPDH. **(B)** Statistical analysis was performed to compare relative protein levels of AIM2, Caspase-1, GSDMD, IL-1β, and IL-18 in PBMCs from healthy controls (n=8, n=5, n=6, n=6, n=6), asymptomatic hyperuricemia patients (n=8, n=7, n=7, n=5, n=7), and acute gouty arthritis patients (n=8, n=7, n=7, n=5, n=8). *P<0.05, **P < 0.01, ***P < 0.001.

**Figure 3** demonstrates that in PBMCs, the levels of proteins such as AIM2, Caspase-1, GSDMD, IL-1β, and IL-18 were markedly elevated in the AGA group when compared to the HC group, with statistical significance (*P*<0.05 for AIM2 and Caspase-1; *P*<0.01 for GSDMD; *P*<0.001 for IL-1β and IL-18). Moreover, the AGA group also displayed a substantial increase in the proteins AIM2, IL-1β, and IL-18 relative to the AHU group, which was statistically significant (*P*<0.05 for each). Conversely, the AHU group showed only a marginal rise in the proteins AIM2, GSDMD, and IL-1β compared to the HC group, which was not statistically significant (*P*>0.05 for all). Nonetheless, the proteins Caspase-1 and IL-18 were present at significantly higher levels in the AHU group compared to the HC group with significant differences (*P*<0.05 for both).

### The concentration of IL-18 and IL-1β in the serum of the three groups

**Figure 4:**
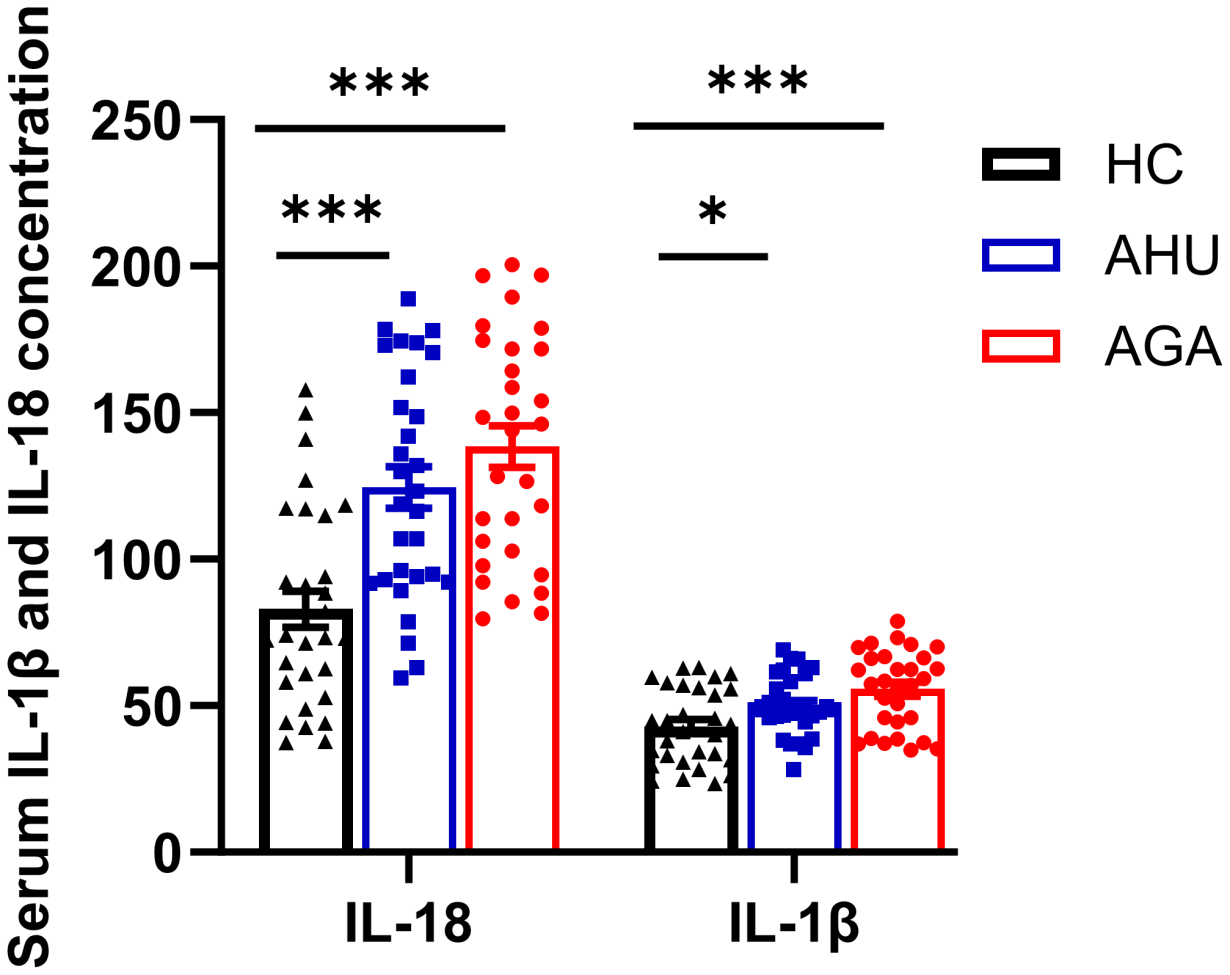
The concentration of IL-1β and IL-18 in serum was assessed by ELISA. There were 30 samples in each group. *P<0.05, ***P < 0.001.

**Figure 4** indicates that the concentrations of IL-18 in the serum for participants in the AGA and AHU cohorts were markedly elevated compared with the HC group, with the difference being statistically significant (*P* < 0.001 for both). Additionally, the same figure shows that the concentrations of IL-1β in the AGA and AHU groups were significantly greater than in the HC group (*P* < 0.001, *P* < 0.05). However, there was no statistical significance in the concentrations of IL-18 or IL-1β when comparing the AGA group to the AHU group (*P* > 0.05 for both).

### Correlation analysis of the level between dsDNA with Clinical characteristics and laboratory indices

Analysis revealed a moderate correlation between dsDNA concentrations and levels of BMI (r = 0.353, *P* = 0.001), TG (r = 0.311, *P* = 0.003), UA (r = 0.371, *P* = 0.000), WBC (r = 0.360, *P* = 0.000), ESR (r = 0.414, *P* = 0.000) and CRP (r = 0.427, *P* = 0.000), and a weak correlation with PLT (r = 0.277, *P* = 0.008), TC (r = 0.242, *P* = 0.022), serum IL-1β (r = 0.271, *P* = 0.010), and serum creatine (r = 0.252, *P* = 0.017) in this research (shown in **Table 2**).

**Table 2:** Correlation analysis of the level between dsDNA with Clinical characteristics and laboratory indices.

		BMI	WBC	Hb	PLT	ESR	CRP	FBG	TC
dsDNA	r	0.353^**^	0.360^**^	0.113	0.277^**^	0.414^**^	0.427^**^	-0.007	0.242^*^
	*P*	0.001	0.000	0.290	0.008	0.000	0.000	0.946	0.022
		TG	UA	ALT	Scr	BUN	Ccr	Serum IL-1β	Serum IL-18
	r	0.311^**^	0.371^**^	0.075	0.252^*^	-0.102	0.118	0.271^**^	0.149
	*P*	0.003	0.000	0.484	0.017	0.337	0.267	0.010	0.160

*P<0.05, **P<0.01.

### Correlation analysis between AIM2 protein and the inflammasome components

**Figure 5:**
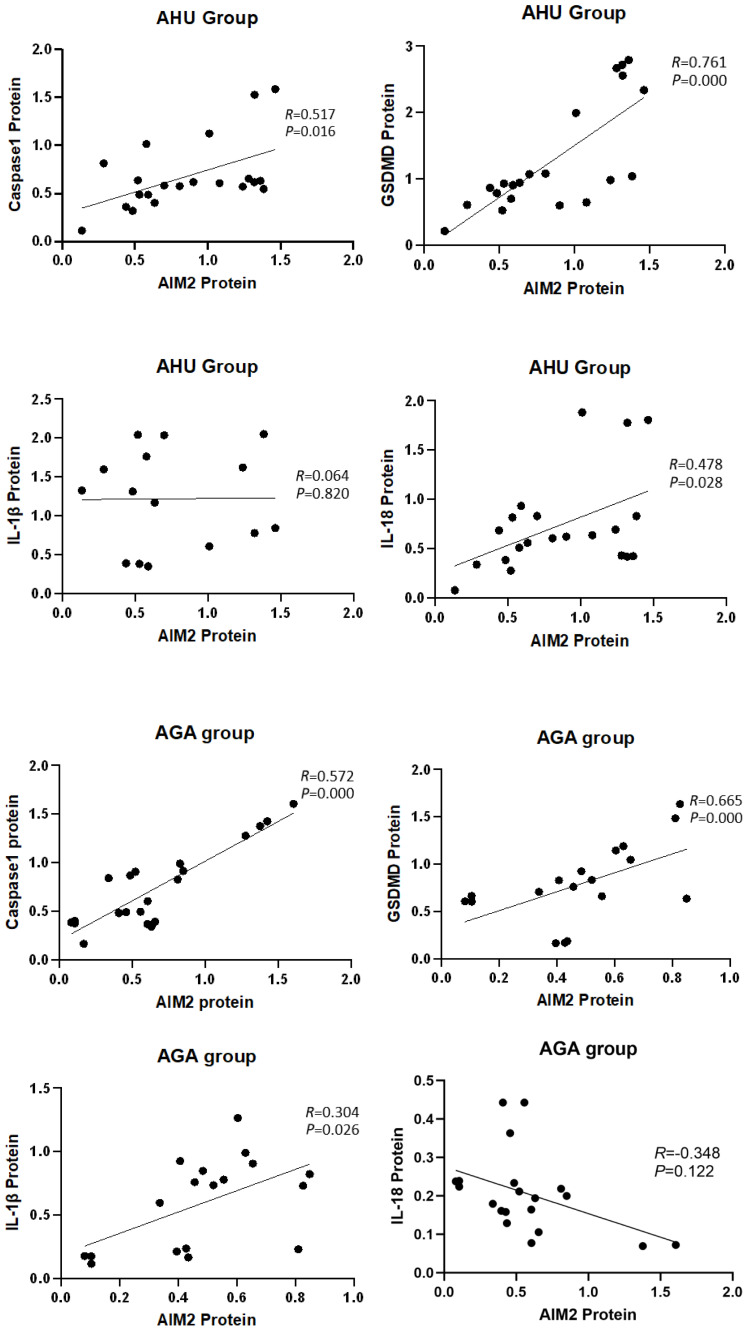
Correlation analysis of the AIM2 protein with inflammasome components and inflammatory factors.

The analysis depicted in **Figure 5** indicated a significant correlation between the expression of AIM2 protein and the levels of Caspase-1 and GSDMD proteins in the AHU (*R* = 0.517, *P* = 0.016; *R* = 0.761, *P* = 0.000) and AGA groups (*R* = 0.572, *P* = 0.000; *R* = 0.665, *P* = 0.000). The expression of AIM2 protein correlated with IL-1β protein in the AGA group (*R* = 0.304, *P* = 0.026). However, the expression of AIM2 protein didn’t correlate with IL-1β protein in the AHU group (*R* = 0.064, *P* = 0.820). In the AHU group, the AIM2 protein demonstrated a statistically significant positive correlation with IL-18 (*R* = 0.478, *P* = 0.028). Conversely, in the AGA group, there was no observed correlation between AIM2 protein and IL-18 (*R* = -0.348, *P* = 0.122).

## Discussion

Double-stranded DNA is the sole ligand of the AIM2 inflammasome. To assess the feasibility of this study, we first measured the plasma levels of double-stranded DNA in the healthy control (HC), AHU, and AGA groups. Plasma DNA is non-cellular DNA circulating in the blood, which can exist as DNA-protein complexes or as free DNA fragments. Due to the release of DNA from a small number of necrotic or apoptotic cells during metabolism, the generation and clearance of DNA are in dynamic equilibrium, resulting in a relatively stable low concentration of free DNA in the blood of healthy individuals. Our findings underscore the impact of obesity on chronic low-grade inflammation (see **Table 1**, **Figure 4**), which may precipitate DNA damage and impair DNA repair mechanisms, as suggested by previous studies (28, 29). The accumulation of DNA damage, particularly in response to high-fat diets (30, 31), is further supported by the presence of γH2AX foci, an early indicator of DNA double-strand breaks (32). Additionally, the correlation between overweight status in adolescents and lymphocytic DNA damage (33) and the association of obesity with various DNA lesions, including double-strand breaks and oxidative base damage, which are reflective of BMI and DNA damage indices (29), align with our observations. In the AHU group, significantly elevated dsDNA levels were observed compared to the HC group (see **Figure 1**), which is consistent with the metabolic syndrome phenotype characterized by obesity, dyslipidemia, and mild inflammation (see **Table 1**, **Figure 4**). Moreover, the AGA group exhibited even higher dsDNA levels (see **Figure 1**), suggesting a more pronounced inflammatory state. Despite no significant differences in metabolic factors between the AGA and AHU groups, the AGA group displayed significantly higher levels of inflammatory markers, such as platelets, ESR, and CRP (see **Table 1**). The moderate correlation between dsDNA levels and these inflammatory markers indicates a potential role for acute inflammation in the elevated dsDNA levels observed in AGA patients (refer to **Table 2**). AGA is recognized as a disease involving a complex interplay within the “metabolic-immune-inflammatory” network, where various inflammatory factors contribute to disease onset and progression (34). The aggregation of neutrophils at crystal deposition sites in AGA and their subsequent release of inflammatory mediators, such as IL-1β and IL-18, initiate a local acute inflammatory response. The activation of IL-1β further stimulates the secretion of neutrophil extracellular traps (NETs), composed of DNA, histones, and granules, which, when in excess or not promptly cleared, can induce the release of damage-associated molecular patterns (DAMPs) and trigger inflammatory responses (35). The detection of dsDNA in the synovial fluid of gout patients (36) and the association of acute gout attacks with increased oxidative stress and mitochondrial dysfunction, leading to the release of mitochondrial DNA into the circulation, further support the role of dsDNA in inflammatory processes. Additionally, the recent discovery that pyroptosis can lead to the loss of membrane integrity and leakage of intracellular contents, potentially contributing to increased dsDNA levels, highlights the complexity of dsDNA dynamics in disease states. In conclusion, the clearance of plasma dsDNA fragments emerges as a promising therapeutic target for AGA.

AIM2 is the sole inflammasome capable of detecting double-stranded DNA. Recent studies using gene-silenced cells and gene-knockout animal models have shown that AIM2 recruits ASC to form an inflammatory complex upon sensing dsDNA fragments of 80-200bp, which in turn activates Caspase-1 and facilitates the maturation and release of inflammatory cytokines such as IL-1β and IL-18 (9, 37). Our understanding of the AIM2 inflammasome’s role in pathogenesis has been furthered by observations of its upregulated expression, alongside that of ASC and Caspase-1, in PBMCs and neutrophils of rheumatoid arthritis patients. This increase correlates with the severity of the disease (25). Additionally, impaired DNase1 activity in the ductal tissues of Sjögren’s syndrome patients and non-tumor salivary gland epithelial cell lines has been implicated in the insufficient degradation of cytoplasmic DNA, leading to the persistent activation of AIM2 (26). Notably, diminished AIM2 expression in a murine lupus model corresponded to improvement in lupus symptoms (38). Discrepancies may exist, however, between the immune mechanisms of AIM2 inflammasome observed under artificial conditions and those in human physiology. Prior studies have reported elevated dsDNA levels in the peripheral blood of systemic lupus erythematosus (SLE) patients, alongside heightened *AIM2* mRNA expression in PBMCs. These findings were associated with kidney involvement and disease activity (24). Su X and colleagues extended this research by examining AIM2 inflammasome expression in Chinese patients with acute and chronic brucellosis (39). Their findings indicate a disease-stage-specific modulation of AIM2 inflammasome components. Acute brucellosis patients exhibit higher AIM2 and lower ASC expression, while chronic brucellosis patients show the opposite pattern. Furthermore, acute brucellosis was associated with significantly higher serum IL-18 levels (39). In our study, we document a pronounced upregulation of *AIM2* mRNA and protein in PBMCs from individuals with AGA and AHU, in contrast to healthy controls (as shown in **Figures 2**, **3**). The AHU group displayed a statistically significant higher concentration of AIM2 mRNA compared to the AGA group (indicated in **Figure 2**). Conversely, the AGA group presented with markedly elevated AIM2 protein levels compared to the AHU group, as shown in **Figure 3**. These findings suggest a nuanced role of AIM2 in the pathogenesis of gout and asymptomic hyperuricemia. NLRP3 is a widely recognized inflammasome and identified as playing a crucial role in the innate immune response associated with gout. Both NLRP3 and AIM2 mediated the canonical pyroptotic pathway. The regulation of NLRP3 and AIM2, as well as the release of pro-IL-1β are associated with NF-κB activation through various receptors, culminating in the transcription of pro-inflammatory cytokine genes and the attraction of inflammatory cells to the joint. Mild-to-moderate pyroptosis can be beneficial, but severe pyroptosis can harm the organism by increasing cell death (40). Our findings suggest that the AIM2 inflammasome responds differently in different states, and the influence of AIM2 on patients with AGA and AHU may be biphasic regulation to protect the host. It deserves more attention to investigate further to clarify the impact of AIM2 inflammasome on AHU and AGA. The interplay of NLRP3 and AIM2 in cellular inflammatory dialogues is an area that requires further investigation.

Caspase-1 as one of the members of the caspase family, functions as the principal effector in the canonical pyroptotic pathway. The canonical pyroptotic pathway, also known as the caspase 1-dependent pathway, involves the activation of inflammasomes such as NLRP3, NLRC4, AIM2, Pyrin, etc. In the molecular pathways of MSU-triggered inflammation, the NLRP3 inflammasome plays a crucial role in the innate immune response associated with gout. The NLRP3 inflammasome depends on a two-signal initiation system, which avoids unregulated activation that would damage the host. The first signal stimulates NF-κB via TLR4 and TLR2, leading to the production of pro-IL-1β, pro-caspase-1, and inflammasome components (41). In the cytoplasm, pro-caspase-1 exists as an inactive zymogen. Monosodium urate crystals act as the second activation signal, causing the assembly of inflammasome and activation of Caspase-1 (42). After activation, pro-caspase-1 is cleaved and separated into its subunits, p20 and p10. These subunits combine to form a functional p20/p10 heterodimeric enzyme, which functions as the activated form of Caspase-1, which proteolyzes pro-IL-1β to mature IL-1β (43). IL-1β then interacts with the IL-1β receptor to trigger a downstream signaling cascade involving proinflammatory cytokines and chemokines, resulting in the recruitment of neutrophils and other cells to the site of crystal deposition. Several regulatory mechanisms are involved in NLRP3 inflammasome activation by MSU crystals, such as NETosis, mitochondrial ROS generation, ATP, K+ efflux, etc (41). Many researches besides our previous investigation revealed a significant upregulation of NLRP3 and Caspase-1 expression in the PBMCs of individuals with AGA and the synovial tissues of rodent AGA analogs (44). AIM2 inflammasome is the first non-NLR family member and belongs to the Caspase-1-dependent canonical pyroptotic pathway. In previous studies, elevated expression of AIM2 and Caspase-1 has been observed in rheumatoid arthritis (25), systemic lupus erythematosus (45), Sjogren’s syndrome (26), and psoriasis (46). AIM2 mRNA or protein expression is associated with Caspase-1 mRNA or protein expression and is related to disease severity. In this study, we observed an increase in the levels of *Caspase-1* mRNA and protein expression not only in the PBMCs of individuals with AGA but also in those with AHU (see **Figures 2**, **3**). Moreover, the expression levels of the Caspase-1 protein positively correlated with the AIM2 protein (as shown in **Figure 5**). These findings suggest a synergistic effect between Caspase-1 expression and AIM2 inflammasome activation in the pathogenesis of AGA. Both NLRP3 and AIM2, as components of the canonical pyroptosis pathways, can activate Caspase-1. What relationship do they have in acute gout attacks and asymptomatic hyperuricemia? Further investigation is warranted.

GSDMD and GSDME are members of the Gasdermin superfamily and are closely associated with the process of pyroptosis (47, 48). GSDMD acts as a substrate of Caspase-1 and participates in inflammatory responses (49). Cleaved by Caspase-1, GSDMD converted into C-terminal and N-terminal domains during pyroptosis. The N-terminal domain can initiate pyroptosis, whereas the C-terminal domain inhibits pyroptosis by binding to the N-terminal domain. Caspase-1 can release GSDMD-N from the inhibitory state of GSDMD-C, which leads to the formation of pores on the cell membrane that are 10–20 nm in diameter. Intracellular substances flow out, and water flows in through these pores, leading to an increase in the osmotic pressure, which causes cell membrane rupture and cell pyroptosis (37, 50). Studies have shown that GSDMD-deficient cells cannot undergo pyroptosis upon stimulation, resulting in a significantly reduced mortality rate post-stimulation, indicating that GSDMD plays a crucial role in pyroptosis (51). Previous studies have also documented similar findings, albeit with the involvement of the NLRP3 inflammasome (52, 53). Accompanied by the inhibition of pyroptosis and reduced IL-1β release, the expression of NLRP3 inflammasome was suppressed both *in vivo* and *in vitro* through knockdown of GSDMD (54). In mice with AGA induced by MSU, GSDMD inhibition via siRNA markedly reduced ankle swelling and inflammation, verified by histopathological analysis (54). In this study, the mRNA and protein levels of the *GSDMD* increased significantly in the AGA group compared with the HC group (see **Figures 2**, **3**). *GSDMD* mRNA and protein levels increased in the AHU group compared to the HC group, but only the mRNA level exhibited significance (see **Figures 2**, **3**). GSDMD protein slightly increased without statistically significant, aligning with the AHU group’s characteristics. There were significantly positive correlations between the protein levels of GSDMD and AIM2 in both AGA and AHU groups. These findings suggested that GSDMD may be involved in AIM2 inflammasome-mediated pyroptosis in patients with AGA and AHU.

The cytokines IL-1β and IL-18 are common pro-inflammatory factors in the IL-1 family. IL-1β is primarily secreted by activated macrophages, which trigger downstream pro-inflammatory cytokines and chemokines by binding to IL-1β receptors. This interaction stimulates the accumulation of neutrophils and other cells at the site of crystal deposition, thus initiating and exacerbating local inflammatory reactions. IL-18 is found in monocytes in the blood of healthy individuals and epithelial cells throughout the gastrointestinal tract. Its secretion is mainly related to Caspase-1, thereby playing a critical role in inflammation and immune regulation (55). Some studies have found that the concentrations of uric acid in the serum of AGA patients and the synovium of model mice positively correlated with the levels of IL-1β and IL-18 (56, 57). Moreover, IL-1β blockers can significantly improve the symptoms of acute gouty arthritis (56, 57). In this study, we found that the levels of IL-1β and IL-18 increased significantly in the sera and PBMCs of patients with AGA, and slightly increased in the patients with AHU compared with healthy volunteers (see **Figures 2**–**4**). The expression of AIM2 protein correlated with IL-1β protein in the AGA group, on the contrary, unrelated to IL-1β protein in the AHU group (as depicted in **Figure 5**). AIM2 protein exhibited a positive correlation with IL-18 in the AHU group and no correlation was observed in the AGA groups (as illustrated in **Figure 5**). This distinction is congruent with the distinct immunopathological characteristics of asymptomatic hyperuricemia and a tendency for acute gouty arthritis spontaneous remission.

To summarize, AGA is a complex disorder characterized by metabolic, immunologic, and inflammatory dysregulation. The AIM2-mediated canonical pathway plays a significant role in the onset and development of AGA and AHU. The modulation of AIM2 inflammasome expression might be a promising target for AGA therapy. Our research has certain limitations, such as a limited sample size, a single-site design, potential selection bias, and neglecting the impact of NLRP3. Therefore, further investigations involving cultivated gene-silenced cells and gene knockout animal models are warranted to elucidate the precise contribution of the AIM2 inflammasome-mediated pyroptosis pathway in AGA and asymptomatic hyperuricemia.

## Data availability statement

The original contributions presented in the study are included in the article/supplementary material. Further inquiries can be directed to the corresponding author.

## Ethics statement

The studies involving humans were approved by Medical Ethics Committee of the General Hospital of Northern Theater Command. The studies were conducted in accordance with the local legislation and institutional requirements. The participants provided their written informed consent to participate in this study.

## Author contributions

JC: Formal analysis, Investigation, Methodology, Software, Writing – original draft. JT: Funding acquisition, Supervision, Writing – review & editing. PL: Conceptualization, Formal analysis, Project administration, Resources, Supervision, Writing – original draft, Writing – review & editing. DF: Data curation, Investigation, Software, Visualization, Writing – original draft. LG: Data curation, Resources, Validation, Visualization, Writing – review & editing. RS: Data curation, Investigation, Validation, Writing – review & editing.

## References

[1] Liu R Han C Wu D Xia XH Gu JQ Guan HX. Prevalence of hyperuricemia and gout in mainland China from 2000 to 2014:A systematic review and meta-analysis. Biomed Res Int. (2015) 2015:762820. doi: 10.1155/2015/762820 26640795 PMC4657091

[2] Dehlin M Jacobsson L Roddy E. Global epidemiology of gout: prevalence, incidence, treatment patterns and risk factors. Nat Rev Rheumatol. (2020) 16:380–90. doi: 10.1038/s41584-020-0441-1 32541923

[3] Dalbeth N Gosling AL Gaffo A Abhishek A. Gout. Lancet. (2021) 397:1843–55. doi: 10.1016/S0140-6736(21)00569-9 33798500

[4] Bursill D Taylor W Terkeltaub R Kuwabara M Merriman TR Grainger R. Gout, hyperuricemia, and crystal-associated disease network consensus statement regarding labels and definitions for disease elements in gout. Arthritis Care Res. (2019) 71:427–34. doi: 10.1002/acr.23607 PMC6252290 29799677

[5] Joosten LAB Crişan TO Bjornstad P Johnson RJ. Asymptomatic hyperuricaemia: a silent activator of the innate immune system. Nat Rev Rheumatol. (2020) 16:75–86. doi: 10.1038/s41584-019-0334-3 31822862 PMC7075706

[6] Johnson RJ Bakris GL Borghi C Chonchol MB Feldman D Lanaspa MA. Hyperuricemia, acute and chronic kidney disease, hypertension, and cardiovascular disease: report of a scientific workshop organized by the National Kidney Foundation. Am J Kidney Dis. (2018) 71:851–65. doi: 10.1053/j.ajkd.2017.12.009 PMC7286363 29496260

[7] Cookson BT Brennan MA. Pro-inflammatory programmed cell death. Trends Microbiol. (2001) 9:113–4. doi: 10.1016/S0966-842X(00)01936-3 11303500

[8] Man SM Karki R Kanneganti TD. Molecular mechanisms and functions of pyroptosis, inflammatory caspases, and inflammasomes in infectious diseases. Immunol Rev. (2017) 277:61–75. doi: 10.1111/imr.12534 28462526 PMC5416822

[9] Sharma BR Karki R Kanneganti TD. Role of AIM2 inflammasome in inflammatory diseases, cancer and infection. Eur J Immunol. (2019) 49:1998–2011. doi: 10.1002/eji.201848070 31372985 PMC7015662

[10] Chai R Li Y Shui L Ni L Zhang A. The role of pyroptosis in inflammatory diseases. Front Cell Dev Biol. (2023) 11:1173235. doi: 10.3389/fcell.2023.1173235 37250902 PMC10213465

[11] Gaidt MM Hornung V. Pore formation by GSDMD is the effector mechanism of pyroptosis. EMBO J. (2016) 35:2167–9. doi: 10.15252/embj.201695415 PMC5069554 27572465

[12] Lu A Magupalli VG Ruan J Yin Q Atianand MK Vos MR. Unified polymerization mechanism for the assembly of ASC-dependent inflammasomes. Cell. (2014) 156:1193–206. doi: 10.1016/j.cell.2014.02.008 PMC4000066 24630722

[13] Broz P Dixit VM. Inflammasomes: mechanism of assembly, regulation, and signaling. Nat Rev Immunol. (2016) 16:407–20. doi: 10.1038/nri.2016.58 27291964

[14] Man SM Kanneganti TD. Regulation of inflammasome activation. Immunol Rev. (2015) 265:6–21. doi: 10.1111/imr.12296 25879280 PMC4400844

[15] Fernandes-Alnemri T Yu JW Datta P Wu J Alnemri ES. AIM2 activates the inflammasome and cell death in response to cytoplasmic DNA. Nature. (2009) 458:509–13. doi: 10.1038/nature07710 PMC2862225 19158676

[16] Hornung V Ablasser A Charrel-Dennis M Bauernfeind F Horvath G Caffrey DR. AIM2 recognizes cytosolic dsDNA and forms a caspase-1-activating inflammasome with ASC. Nature. (2009) 458:514–8. doi: 10.1038/nature07725 PMC2726264 19158675

[17] Hu B Jin C Li HB Tong J Ouyang X Cetinbas NM. The DNA-sensing AIM2 inflammasome controls radiation-induced cell death and tissue injury. Science. (2016) 354:765–8. doi: 10.1126/science.aaf7532 PMC5640175 27846608

[18] Lian Q Xu J Yan S Huang M Ding H Sun X. Chemotherapy-induced intestinal Inflammatory Responses are Mediated by Exosome Secretion of Double-Strand DNA via AIM2 Inflammasome Activation. Cell Res. (2017) 27:784–800. doi: 10.1038/cr.2017.54 28409562 PMC5518874

[19] Yogarajah T Ong KC Perera D Wong KT. AIM2 inflammasome-mediated pyroptosis in enterovirus A71-infected neuronal cells restricts viral replication. Sci Rep. (2017) 7:5845. doi: 10.1038/s41598-017-05589-2 28724943 PMC5517550

[20] Lugrin J Martinon F. The AIM2 inflammasome: sensor of pathogens and cellular perturbations. Immunol Rev. (2018) 281:99–114. doi: 10.1111/imr.12618 29247998

[21] Moriyama M Nagai M Maruzuru Y Koshiba T Kawaguchi Y Ichinohe T. Influenza virus-induced oxidized DNA activates inflammasomes. iScience. (2020) 23:101270. doi: 10.1016/j.isci.2020.101270 32592999 PMC7293844

[22] Jin T Perry A Jiang J Smith P Curry JA Unterholzner L. Structures of the HIN domain: DNA complexes reveal ligand binding and activation mechanisms of the AIM2 inflammasome and IFI16 receptor. Immunity. (2012) 36:561–71. doi: 10.1016/j.immuni.2012.02.014 PMC3334467 22483801

[23] Matyszewski M Morrone SR Sohn J. Digital signaling network drives the assembly of the AIM2-ASC inflammasome. Proc Natl Acad Sci USA. (2018) 115:E1963–e1972. doi: 10.1073/pnas.1712860115 29440442 PMC5834677

[24] Choubey D Panchanathan R. Absent in melanoma 2 proteins in SLE. Clin Immunol. (2017) 176:42–8. doi: 10.1016/j.clim.2016.12.011 PMC5346332 28062222

[25] Mendez-Frausto G Medina-Rosales MN Uresti-Rivera EE Baranda-Cándido L Zapata-Zúñiga M Bastián Y. Expression and activity of AIM2-inflammasome in rheumatoid arthritis patients. Immunobiology. (2020) 225:151880. doi: 10.1016/j.imbio.2019.11.015 31836304

[26] Vakrakou AG Svolaki IP Evangelou K Gorgoulis VG Manoussakis MN. Cell-autonomous epithelial activation of AIM2 (Absent in melanoma-2) inflammasome by cytoplasmic DNA accumulations in primary sjögren’s syndrome. J Autoimmun. (2020) . 108:102381. doi: 10.1016/j.jaut.2019.102381 31919014

[27] Neogi T Jansen TL Dalbeth N Fransen J Schumacher HR Berendsen D. 2015 Gout classification criteria: an American College of Rheumatology/European League Against Rheumatism collaborative initiative. Ann Rheum Dis. (2015) 74:1789–98. doi: 10.1136/annrheumdis-2015-208237 PMC4602275 26359487

[28] Kay J Thadhani E Samson L Engelward B. Inflammation-induced DNA damage, mutations and cancer. DNA Repair (Amst). (2019) 83:102673. doi: 10.1016/j.dnarep.2019.102673 31387777 PMC6801086

[29] Wlodarczyk M Nowicka G. Obesity, DNA damage, and development of obesity-related diseases. Int J Mol Sci. (2019) 20:1146. doi: 10.3390/ijms20051146 30845725 PMC6429223

[30] Zwamborn RA Slieker RC Mulder PC Zoetemelk I Verschuren L Suchiman HE. Prolonged high-fat diet induces gradual and fat depot-specific DNA methylation changes in adult mice. Sci Rep. (2017) 7:43261. doi: 10.1038/srep43261 28256596 PMC5335669

[31] Keleher MR Zaidi R Hicks L Shah S Xing XY Li DF. A high-fat diet alters genome-wide DNA methylation and gene expression in SM/J mice. BMC Genomics. (2018) 19:888. doi: 10.1186/s12864-018-5327-0 30526554 PMC6286549

[32] Redon CE Nakamura AJ Martin OA Parekh PR Weyemi US Bonner WM. Recent developments in the use of γ -H2AX as a quantitative DNA double strand break biomarker. Aging (Albany NY). (2011) 3:168–74. doi: 10.18632/aging.v3i2 PMC3082012 21325706

[33] Usman M Woloshynowych M Britto JC Bilkevic I Glassar B Chapman S. Obesity, oxidative DNA damage and vitamin D as predictors of genomic instability in children and adolescents. Int J Obes (Lond). (2021) 45:2095–107. doi: 10.1038/s41366-021-00879-2 PMC8380542 34158611

[34] Bodofsky S Merriman TR Thomas TJ Schlesinger N. Advances in our understanding of gout as an auto-inflammatory disease. Semin Arthritis Rheum. (2020) 50:1089–100. doi: 10.1016/j.semarthrit.2020.06.015 32916560

[35] Vedder D Gerritsen M Duvvuri B van Vollenhoven RF Nurmohamed MT Lood C. Neutrophil activation identifies patients with active polyarticular gout. Arthritis Res Ther. (2020) 22:148. doi: 10.1186/s13075-020-02244-6 32552822 PMC7304179

[36] Mitroulis I Kambas K Ritis K. Neutrophils, IL- 1β, and gout: is there a link? Semin Immunopathol. (2013) 35:501–12. doi: 10.1007/s00281-013-0361-0 23344781

[37] Broz P Pelegrin P Shao F. The gasdermins are a protein family executing cell death and inflammation. Nat Rev Immunol. (2020) 20:143–57. doi: 10.1038/s41577-019-0228-2 31690840

[38] Yang M Long D Hu L Zhao Z Li Q Guo Y. AIM2 deficiency in B cells ameliorates systemic lupus erythematosus by regulating Blimp-1-Bcl-6 axis-mediated B-cell differentiation. Signal Transduct Target Ther. (2021) 6:341. doi: 10.1038/s41392-021-00725-x 34521812 PMC8440614

[39] Su X Zhao SG Song YJ. Expression of NLRP3 and AIM2 inflammasome in Peripheral blood in Chinese patients with acute and chronic brucellosis. Sci Rep. (2022) 12:15123. doi: 10.1038/s41598-022-19398-9 36068262 PMC9448728

[40] Lu F Lan Z Xin Z He C Guo Z Xia X. Emerging insights into molecular mechanisms underlying pyroptosis and functions of inflammasomes in diseases. J Cell Physiol. (2020) 235:3207–21. doi: 10.1002/jcp.29268 PMC13482838 31621910

[41] He Y Hara H Núñez G. Mechanism and regulation of NLRP3 inflammasome activation. Trends Biochem Sci. (2016) 41:1012–21. doi: 10.1016/j.tibs.2016.09.002 PMC5123939 27669650

[42] So AK Martinon F. Inflammation in gout: mechanisms and therapeutic targets. Nat Rev Rheumatol. (2017) 13:639–47. doi: 10.1038/nrrheum.2017.155 28959043

[43] Boucher D Monteleone M Coll RC Chen KW Ross CM Teo JL. Caspase-1 self-cleavage is an intrinsic mechanism to terminate inflammasome activity. J Exp Med. (2018) 215:827–40. doi: 10.1084/jem.20172222 PMC5839769 29432122

[44] Tian J Wang BC Xie B Liu X Zhou D Hou X. Pyroptosis inhibition alleviates potassium oxonate- and monosodium urate-induced gouty arthritis in mice. Mod Rheumatol. (2022) 32:221–30. doi: 10.1080/14397595.2021.1899569 33705241

[45] Shin JI Lee KH Joo YH Lee JM Jeon J Jung HJ. Inflammasomes and autoimmune and rheumatic diseases: A comprehensive review. J Autoimmun. (2019) .103:102299. doi: 10.1016/j.jaut.2019.06.010 31326231

[46] Dombrowski Y Peric M Koglin S Kammerbauer C Göß C Anz D. Cytosolic DNA triggers inflammasome activation in keratinocytes in psoriatic lesions. Sci Transl Med. (2011) 3:82ra38. doi: 10.1126/scitranslmed.3002001 PMC3235683 21562230

[47] Qiu S Liu J Xing F. 'Hints'in the killer protein gasdermin D: unveiling the secrets of gasdermins driving cell death. Cell Death Differ. (2017) 24:588–96. doi: 10.1038/cdd.2017.24.2020.02.002 PMC5384029 28362726

[48] Fischer FA Chen KW Bezbradica JS. Posttranslational and therapeutic control of gasdermin-mediated pyroptosis and inflammation. Front Immunol. (2021) 12:661162. doi: 10.3389/fimmu.2021.661162 33868312 PMC8050342

[49] Shi J Zhao Y Wang K Shi X Wang Y Huang H. Cleavage of GSDMD by inflammatory caspases determines pyroptotic cell death. Nature. (2015) 526:660–5. doi: 10.1038/nature15514 26375003

[50] Wang K Sun Q Zhong X Zeng M Zeng H Shi X. Structural mechanism for GSDMD targeting by autoprocessed caspases in pyroptosis. Cell. (2020) 180:941–55. doi: 10.1016/j.cell 32109412

[51] Bergsbaken T Fink SL Cookson BT. Pyroptosis: host cell death and inflammation. Nat Rev Microbiol. (2009) 7:99–109. doi: 10.1038/nrmicro2070 19148178 PMC2910423

[52] Martinon F Petrilli V Mayor A Tardivel A Tschopp J. Gout-associated uric acid crystals activate the NALP3 inflammasome. Nature. (2006) 440:237–41. doi: 10.1038/nature04516 16407889

[53] Hao K Jiang W Zhou M Li H Chen Y Jiang F. Targeting BRD4 prevents acute gouty arthritis by regulating pyroptosis. Int J Biol Sci. (2020) 16:3163–73. doi: 10.7150/ijbs.46153 PMC7645998 33162822

[54] Ye SM Zhou MZ Jiang WJ Liu CX Zhou ZW Sun MJ. Silencing of Gasdermin D by siRNA-Loaded PEI-Chol Lipopolymers Potently Relieves Acute Gouty Arthritis through Inhibiting Pyroptosis. Mol Pharm. (2021) 18:667–78. doi: 10.1021/acs.molpharmaceut.0c00229 32579365

[55] Kaplanski G. Interleukin-18: Biological properties and role in disease pathogenesis. Immunol Rev. (2018) 281:138–53. doi: 10.1111/imr.12616 PMC7165732 29247988

[56] Han W Chao H Xie J Yang C Zhao Y Guo Y. Doliroside A attenuates monosodium urate crystal-induced inflammation by targeting NLRP3 inflammasome. Eur J Pharmacol. (2014) 740:321–8. doi: 10.1016/j.ejphar.2014.07.023 25064339

[57] Tian J Zhou D Xiang L Liu X Zhang H Wang B. miR-223–3p inhibits inflammation and pyroptosis in monosodium urate-induced rats and fibroblast-like synoviocytes by targeting NLRP3. Clin Exp Immunol. (2021) 204:396–410. doi: 10.1111/cei.13587 33608866 PMC8119838

